# A Near-Infrared Optical Tomography System Based on Photomultiplier Tube

**DOI:** 10.1155/2007/28387

**Published:** 2007-07-12

**Authors:** Huacheng Feng, Jing Bai, Xiaolei Song, Gang Hu, Junjie Yao

**Affiliations:** Department of Biomedical Engineering, Tsinghua University, Beijing 100084, China

## Abstract

Diffuse optical tomography (DOT) is a rapidly growing discipline in recent years. It plays an important role in many fields, such as detecting breast cancer and monitoring the cerebra oxygenation. In this paper, a relatively simple, inexpensive, and conveniently used DOT system is presented in detail, in which only one photomultiplier tube is employed as the detector and an optical multiplexer is used to alter the detector channels. The 32-channel imager is consisted of 16-launch fibers and 16-detector fibers bundles, which works in the near-infrared (NIR) spectral range under continuous-wave (CW) model. The entire imaging system can work highly automatically and harmoniously. Experiments based on the proposed imaging system were performed, and the desired results can be obtained. The experimental results suggested that the proposed imaging instrumentation is effective.

## 1. INTRODUCTION

Optical imaging is a traditional imaging technique for medical purpose [1]. However, diffuse optical tomography (DOT) is a relatively new discipline and drew increasing interest in recent years [2, 3]. If the target-specific fluorescent contrast agent is employed in DOT, it can probe molecular event in vivo [4–6], which is very useful to detect disease in its early stage, comprehensively understand disease mechanism, and develop new drugs. DOT has many advantages over the conventional imaging techniques. For example, it is not harmful to tissue due to its noninvasive and nonionizing characteristics. Thus it can be repeatedly even continuously used on patients at the bedside. In addition, DOT instrumentation is relatively inexpensive and can be made portable. DOT technique has shown its powerful potential in clinical applications. Currently, its two main applications are monitoring cerebral blood volume and oxygenation and screening breast cancer [7–12].

For neonates, the deficiencies of cerebral blood flow or oxygen may lead to severe irreversible damages to the brain development. The premature babies are more subject to have the risk of cerebral hemorrhage [10]. However, the existing conventional medical imaging modalities are not capable of monitoring the cerebral blood volume and oxygenation continuously without invasion and damage.

Besides, currently the most commonly used conventional means to detect breast tumor is X-ray. It is not suitable to be used on patients continuously or even frequently due to its radiative nature. In addition, when the tumor can be “seen” by the X-ray instrumentation, it is generally too late to be treated.

The optical tomography is a very powerful complementary tool to the existing conventional imaging techniques in the above mentioned fields [13].

Many investigators have contributed considerably to DOT technique, and many excellent DOT systems for medical purpose have been developed [1, 14, 15].

In this paper, we present a DOT imaging system that is based on photomultiplier tube (PMT). In the entire imaging system, only one PMT was employed as the detector and an optical multiplexer was used to alter the detector channels, so that the entire imaging system is relatively compact. Compared to the charge-coupled-device- (CCD-) based imaging system, it is relatively simple and considerably inexpensive. Besides, the proposed imaging instrumentation was designed as a highly automatically system, of which all the components can work harmoniously. In the following discussions, the system principle, including hardware setup and control and data acquisition software, is depicted in detail. Some experiments based on the proposed imaging system were performed to test it. The experimental results demonstrate that the proposed imaging instrumentation is effective.

## 2. THEORETICAL BACKGROUND

The DOT imaging systems can be broadly divided into three categories [2]: continue-wave (CW) system, time-domain (TD) instrumentation, and frequency-domain (FD) modality. Each category has its advantages and disadvantages. In this paper, a CW system is represented in detail, in which the source light is sinusoidally modulated at the frequency of 5 kHz to facilitate the signal processing, such as elimination of noise.

As photons propagate in tissue, they experience scattering as well as absorption. In the near-infrared (NIR) spectral range, scattering is the dominant interaction. The transport process of photons in tissue can be well described by the radiation transport equation (RTE). Under certain assumptions, the RTE can be approximated by the diffusion equation (DE), a partial differential equation [3, 13, 16], which is more commonly used to model light transport in tissue. The diffusion equation in time-domain and frequency-domain has been derived in detail in earlier literature [13]. In the CW case, the DE can be written as

(1) $$-\nabla \left(D\left(\text{r}\right)\nabla \text{Φ}\left(\text{r}\right)\right)+\mu_{a}\left(\text{r}\right)\text{Φ}\left(\text{r}\right)=q\left(\text{r}\right)$$,

where **r** is the location in tissue domain Ω, Φ(**r**) is the photon density distribution, *μ*_*a*_(**r**) is the absorption coefficient distribution, *q*(**r**) is the source term, *D* is the diffusion coefficient given by *D* = 1/[3(*μ*_*a*_ + *μ*′_*s*_)], where *μ*′_*s*_ = (1 − *g*)*μ*_*s*_ is the reduced scattering coefficient, *μ*_*s*_ is the scattering coefficient, and *g* is the anisotropic factor. The spatially dependent diffusion coefficient *D*(**r**) and absorption coefficient *μ*_*a*_(**r**) are the two main optical properties that reflect the function of the diseased and healthy tissues, and generally are the objectives to be reconstructed in DOT.

If the source *q*(**r**) is a collimated incident beam, it can be treated as a “point source” under the surface ∂Ω at a depth of one mean free length [17]. In this situation, *q*(**r**) = *q*_0_ *δ*(**r** − **r**_*s*_), where **r**_*s*_ is the location of the equivalent point source and *q*_0_ is the strength of the source term.

The Robin boundary condition in the steady-state case is usually employed [18]. So the measured quantity on the boundary is expressed as

(2) $$\Gamma \left(\xi \right)=-D\left(\xi \right)\frac{\partial \text{Φ}\left(\xi \right)}{\partial \text{n}}$$,

where **n** is the outward normal at the site *ξ* on the boundary ∂Ω, and Γ(*ξ*) is the measurement photon flux. In the CW case, along with the boundary condition (2), (1) is the most commonly used forward model for DOT and it is also employed in this paper in succeeding discussions.

In CW case, the absorption and diffusion coefficients cannot be recovered simultaneously [19, 20]. When scattering is the dominant interaction, it is absorption coefficient rather than scattering coefficient that often derives the important physiological information [1]. So the spatially dependent absorption coefficient distribution is the main optical property of tissue to be recovered.

The light in the NIR region of 650 nm–900 nm is most commonly used in practical applications [21]. In this spectral range, the principal absorbers, water, lipids, and hemoglobin have their lowest absorption coefficients, and then the penetration depth of the light in tissue is highest [7].

## 3. MATERIALS AND METHODS

### 3.1. System hardware setup

The proposed imaging instrumentation is primarily consisted of optical components, electrical components, control and data acquisition routines, and image reconstruction program. In this section, we present the scheme of the imaging system and its hardware setup.

**Figure 1:**
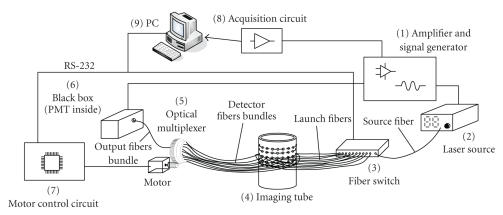
System scheme.

The scheme of the DOT system can be illuminated in Figure 1.

**Figure 2:**
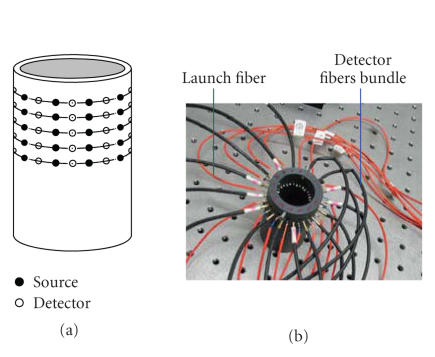
Imaging tube.

The signal generator circuit (1) (homemade) generates a sinusoidal signal at the frequency of 5 kHz, which is used to modulate the intensity of the source light. The laser source (2) (VA671-200, Viasho, China) produces the source light at wavelength of 671 nm with maximum power of 200 mW. The output power of the source light can be adjusted to the desired level by adjusting the current of the laser generator. The purpose to modulate the source light is to facilitate the elimination of noise in succeeding signal processing. The sinusoidally intensity-modulated source light is also named as “AC light” (similarly, the constant intensity light is named as “DC light”), which is guided into a 1 × 16 fiber switch (3) (SUN-FSW 1 × 16 MM, SUN, China) through a source fiber. The AC light is then switched into one of the 16 launch fibers sequentially. The launch fibers are held in the imaging tube (4) (homemade), and launch the source light onto the tissue surface at different site sequentially. The imaging tube is illustrated in detail in Figure 2.

As illustrated in Figure 2, the imaging tube has five rings of bores. On each ring there are 32 bores, of which 16 bores are used to hold the launch fibers and the other 16 bores are for detector fibers bundles. They are separated uniformly. When the launch fibers and the detector fibers bundles are held on the same ring, they are generally used to image in two dimensions (2D). When held in different rings, they are used to image in three dimensions (3D).

The photons that are launched into the tissue undergo scattering and absorption. Some will “quench” when they are absorbed by tissue. The others will “escape” out of the tissue surface after they experience multiplying scattering.

**Figure 3:**
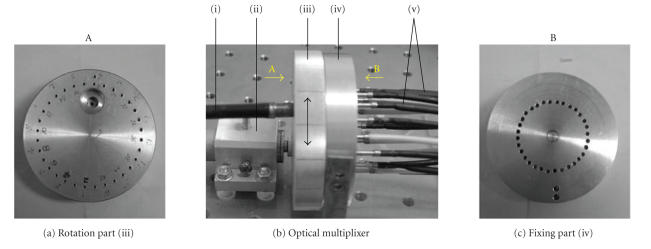
Optical multiplexer. For details, see the text.

The light that transilluminates from tissue is collected by the 16 detector fibers bundles, and then is guided into the optical multiplexer (5), which switches the 16 detector fibers bundles sequentially to the output fibers bundle. The optical multiplexer is homemade, and its principle is similar to the fiber switch (3). The 16 detector fibers bundles have large inner diameter of 1 mm, so that they can collect photons efficiently. However, they are not suitable to be coupled into the fiber switch, because for fiber switch the inner diameter of the coupled fiber is generally required at the *μ*m level, such as 62.5 *μ*m, a very widely used standard of fiber diameter. That is the reason why we used an optical multiplexer rather than a fiber switch to alter the detector channels. The principle of the optical multiplexer is illustrated in Figure 3.

As illustrated in Figure 3, the optical multiplexer has mainly three parts: the motor (ii), the rotation part (iii), and the fixing part (iv). The rotation part and the fixing part are on-axis and coupled through an axletree. The fixing part has 32 bores and in which 16 bores are used to hold the detector fibers bundles (v). The rotation part has one bore that is used to hold the output fibers bundle (i). Driven by the motor, the rotation part can revolve around its axis while the fixing part is fixedly mounted on the platform. When the rotation part rotates to different location, the output fibers bundle will aim at different detector fibers bundles, and then the collected photons can be switched from one of the detector fibers bundles to the output fibers bundle. The inner diameter of the detector fibers bundles is 1 mm and that of the output fibers bundle is 2 mm. So the energy of the light can be guided efficiently into the black box (6) (homemade), as illustrated in Figure 1. Inside the black box there is a photomultiplier tube (PMT: R928, Hamamatsu, Japan), which translates the light to electrical signal. After being amplified and preprocessed, the electrical signal is then sampled into a personal computer (9) by the data acquisition circuit (8) (NI5112, Ni America, Tex, USA) as the raw data to be used to reconstruct the image.

**Figure 4:**
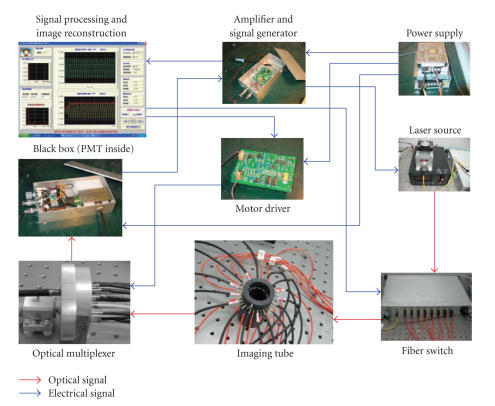
The practical imaging system.

The photos of the practical imaging system are shown in Figure 4.

### 3.2. Instrumentation control and data acquisition software

The primary duties of the instrumentation control and data acquisition software are to sample the raw data, postprocess the data, and control the hardware (fiber switch and the motor of the optical multiplexer). All these functions are integrated together for highly automatical purpose.

The control software is developed by C++ computer language and runs under Windows XP. The process of control and data acquisition can be described as follows: the personal computer delivers a command to the fiber switch by RS-232 serial interface to alter its channels (namely source channels) sequentially, and then the source light is switched into different launch fiber to illuminate different site on the tissue surface. Once the source channel is changed, the computer then delivers a commands to the motor control circuit (7) (as illustrated in Figure 1) to drive the motor, and then drive the rotation part of the optical multiplexer revolved to alter the detector channels. Thus the 16 detector fibers bundles are switched sequentially into the output fibers bundle, and then the light is guided into the black box to illuminate the PMT. The signal translated by PMT is a modulated signal, which is contaminated by the noise, such as the environmental light and the dark current of the PMT. We have two methods to improve the signal quality. One method is that the lock-in amplifier is employed, in which the sinusoidal signal produced by the signal generator is employed as the reference signal, and the amplified signal derived from the PMT as the input signal. Another method is that the digital filter is employed in the signal postprocessing routine in the computer. When the digital filter is used, as the signal is modulated at the frequency of 5 kHz, we employ one digital band-pass filter with the central frequency of 5 kHz to eliminate the noise. Through the digital filter, a relatively “pure” sinusoidal signal can be obtained, and by Hilbert transform, the amplitude, that is, the envelope of the modulated signal, can be extracted (the result of the Hilbert transform of the sinusoidal signal is shown in Figure 6(a)). The arithmetical average of the amplitude is evalued as the raw data to reconstruct the image. Repeat above processes until all source channels and all detector channels have a turn. All the processes are implemented harmoniously and automatically through the instrumentation control and data acquisition software.

**Figure 5:**
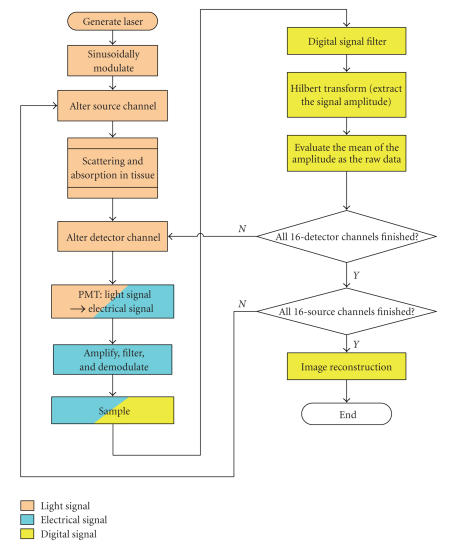
Flow chart of data acquisition and control processes.

The flow chart of above processes and the corresponding signal format of each stage can be illustrated in Figure 5.

**Figure 6:**
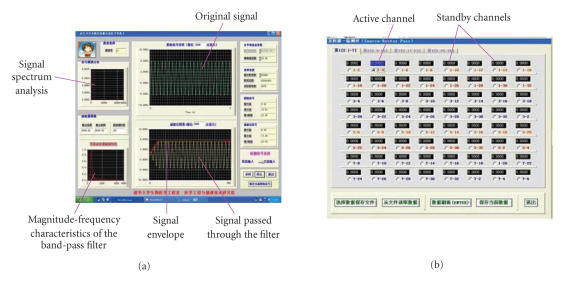
Data acquisition and signal processing GUI.

The graphic user interface (GUI) of the data acquisition and signal processing software is shown in Figure 6. The data acquisition, signal spectrum analysis, and signal processing windows are shown in Figure 6(a), in which the parameters of the digital band-pass filter, such as the cutoff frequency and the order of the filter can be set manually according to the result of the signal spectrum analysis. The source-detector pair value display panel is shown in Figure 6(b), in which the 16 sources and 16 detectors constitute 256 source-detector pairs and they are divided into four pages. The active channel displays the value of current source-detector pair.

### 3.3. Image reconstruction algorithm

In this work, a gradient-based optimization inversion method is used for the absorption coefficient inversion with finite element method solving the forward model [17, 22]. Considering an experimental setting that includes *S* point excitation light sources located at *ξ*_*j*_ ∈ ∂Ω (*j* = 1, …, *S*), and *M*_*j*_ measurement positions *ζ*_*j*,*i*_ ∈ ∂ Ω (*i* = 1, …, *M*_*j*_) for each source *j*, the following objective function can be defined:

(3) $$E=\frac{1}{2}\sum_{j=1}^{S}\sum_{i=1}^{M_{j}}{\left({\left({\text{Γ}}_{j,i}\right)}_{\text{mea}}-{\left({\text{Γ}}_{j,i}\right)}_{c}\right)}^{2}$$,

where Γ_*j*,*i*_ represents the photon intensity measured at position *ζ*_*j*,*i*_ with the incident excitation source located at *ξ*_*j*_. The subscript *c* denotes the values calculated by the forward simulation and mea represents the experimental values.

In practice, the attenuations of launch fibers are inconsistent. So do that of the detector fibers bundles. It means that calibration should be performed to eliminate the effect of the inconsistent attenuations of fibers or fibers bundles. In order to avoid the calibration procedure, in this paper, two sets of data are sampled for relative image reconstruction. One is acquired before the absorber is embedded inside the intralipid. The corresponding measurement is (Γ_*j*,*i*_)_bef_. The other is acquired after the absorber is immersed into the intralipid and the corresponding measurement is (Γ_*j*,*i*_)_aft_. The measurements (Γ_*j*,*i*_)_mea_ in (3) and following equations are given by the formula (Γ_*j*,*i*_)_mea_ = (Γ_*j*,*i*_)_aft_/(Γ_*j*,*i*_)_bef_, which are relative quantities. So the calculated values (Γ_*j*,*i*_)_*c*_ are also relative quantities.

In our work, conjugate gradient (CG) method is used to minimize the objective function. First, the gradient of the objective function needs to be calculated as follow:

(4) $$\nabla E=\sum_{j=1}^{S}\sum_{i=1}^{M_{j}}\left({\left({\text{Γ}}_{j,i}\right)}_{\text{mea}}-{\left({\text{Γ}}_{j,i}\right)}_{c}\right)\cdot \left(-\frac{\partial {\left({\text{Γ}}_{j,i}\right)}_{c}}{\partial \mu_{a}}\right)$$.

Therefore, the gradient vector $\vec{z}$ can be presented as

(5) $$\vec{z}=\nabla E=-{\text{J}}^{T}\text{b}$$,

where **J** is an *M*_TOT_ × *N*_TOT_ Jacobian matrix, $M_{\text{TOT}}=\sum_{j=1}^{S}M_{j}$ is the total measurement number at the boundary, and *N*_TOT_ is the number of the coefficients to be reconstructed. Here **b** is the residual error between the boundary measurements and computation values. Then **J** can be calculated by an adjoint source scheme based on the establishment of PMDF (photon measurement density function, as defined in [23]).

With the gradient calculated, the next step is to conduct one-dimension search in order to find the best step length on this gradient direction. Then, we refresh the absorption coefficient and recalculate the gradient to form iteration computing until the error reaches the supposed value.

## 4. RESULTS AND DISCUSSION

**Figure 7:**
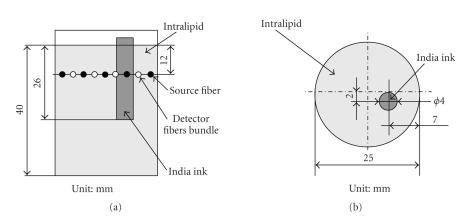
An experimental model.

To test the proposed imaging system, some experiments were performed, of which one model is illustrated in Figure 7.

In the experiment, a glass cup was filled with 1% intralipid, a tissue-like medium. The cup was mounted in the imaging tube. The intralipid is a homogeneous medium, namely, its anisotropic factor *g* = 0. A glass tube of India ink was employed as the heterogeneous object, that is, the simulated absorber. The launch fibers and the detector fibers bundles were held in the imaging tube on the same ring, and they were separated uniformly. Two sets of data were acquired for relative image reconstruction. They were sampled, respectively, before and after the India ink was embedded inside the intralipid. Their geometries and positions are illustrated in Figure 7.

**Figure 8:**
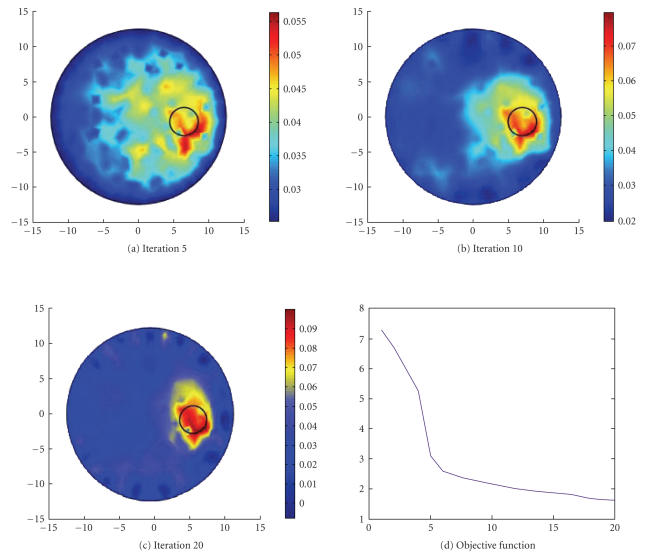
Reconstruction results.

The reconstruction results are illustrated in Figure 8. In Figure 8(a) to Figure 8(c), the circle inside the domain Ω stands for the true size and position of the absorber. We can see, as the iterations increase, the reconstructed result converges gradually to its true solution. The curve of the objective function (3) is shown in Figure 8(d). The other models used to test the imaging system can also derive the desired results. These experimental results suggest that the proposed imaging system is effective.

To solve the DOT is a typical inverse problem. Inverse problem is intrinsically ill-posed, which means that the solution to the problem may not exist (existence) or is not unique (uniqueness), or does not depend continuously on the data (stability) [24, 25]. For a practical physics problem, the existence and uniqueness of the solution can be satisfied naturally or be enforced by mathematical measures. So the stability is the most important profile. If a problem lacks the property of stability, a little of fluctuation of the measured data may lead to the solution deviated significantly from its true solution. To reduce the ill-posedness, regularizations strategies, such as Tikhonov regularization and Landweber iteration, are often employed [24]. However it is essentially expected that the noise polluted the measured data is as small as possible, while it is unavoidable. So in the next generation of the imaging instrumentation, some measures would be taken to eliminate to a great extent the effect of noise. For example, cooling system is employed on the PMT to reduce the dark current.

To understand the model of the noise is very useful to eliminate its influence by using appropriate algorithm. In the imaging instrumentation there are mainly three kinds of noise: thermal noise, shot noise, and relative intensity noise [26]. Usually the shot noise is the principal noise in the imaging system, which mainly rises from the dark current of the photodetector. The shot noise statistics has its origin in Poisson statistics [27]. When the current is significantly large, it is governed by the Gaussian distribution. In this case, the statistical method, such as Bayesian framework, is suitable for the inverse problem [28, 29].

In addition, solving DOT is also a highly underdetermined problem, since the number of the measured data is much less than that of the pixels to be reconstructed. In above experiment, the forward problem was solved by the finite element method (FEM) software and the tissue domain Ω was divided into 1644 elements. However, there are only 16 sources and 16 detectors, namely, 256 known data are available. The number of the known data is much less than that of the elements. It means that the problem is considerably underdetermined. The underdetermining nature is one of the main factors that influences the quality of the reconstructed image, especially the spatial resolution. As noted in the literature [30]: A lack of information cannot be remedied by any mathematical trickery! The most important way to improve the quality of the image is to obtain prior information as more as possible, for example, acquire more data, or take advantage of the anatomical imaging or the physiology information in the reconstruction process.
